# A Unique Capsule Locus in the Newly Designated Actinobacillus pleuropneumoniae Serovar 16 and Development of a Diagnostic PCR Assay

**DOI:** 10.1128/JCM.02166-16

**Published:** 2017-02-22

**Authors:** Janine T. Bossé, Yanwen Li, Rita Sárközi, Marcelo Gottschalk, Øystein Angen, Katerina Nedbalcova, Andrew N. Rycroft, László Fodor, Paul R. Langford

**Affiliations:** a Section of Paediatrics, Department of Medicine, Imperial College London, St. Mary's Campus, London, United Kingdom; b Department of Microbiology and Infectious Diseases, University of Veterinary Medicine, Budapest, Hungary; c Groupe de Recherche sur les Maladies Infectieuses du Porc, Faculté de Médecine Vétérinaire, Université de Montréal, Montréal, Québec, Canada; d Department of Microbiology and Infection Control, Statens Serum Institut, Copenhagen, Denmark; e Veterinary Research Institute, Brno, Czech Republic; f Department of Pathology and Pathogen Biology, The Royal Veterinary College, Hawkshead Campus, Hatfield, United Kingdom; University of Tennessee

**Keywords:** Actinobacillus pleuropneumoniae, PCR, diagnostics, serovar 16

## Abstract

Actinobacillus pleuropneumoniae causes pleuropneumonia, an economically significant lung disease of pigs. Recently, isolates of A. pleuropneumoniae that were serologically distinct from the previously characterized 15 serovars were described, and a proposal was put forward that they comprised a new serovar, serovar 16. Here we used whole-genome sequencing of the proposed serovar 16 reference strain A-85/14 to confirm the presence of a unique capsular polysaccharide biosynthetic locus. For molecular diagnostics, primers were designed from the capsule locus of strain A-85/14, and a PCR was formulated that differentiated serovar 16 isolates from all 15 known serovars and other common respiratory pathogenic/commensal bacteria of pigs. Analysis of the capsule locus of strain A-85/14 combined with the previous serological data show the existence of a sixteenth serovar—designated serovar 16—of A. pleuropneumoniae.

## INTRODUCTION

Actinobacillus pleuropneumoniae is an economically important pathogen in the swine industry, causing peracute to chronic pleuropneumonia (1, 2). Isolates can be classified into two biovars depending on their requirement for NAD, with the majority of isolates belonging to biovar I (require NAD for growth) and biovar II isolates (NAD independent) less frequently identified (3). Isolates can be further differentiated into serovars, based mainly on capsular polysaccharide (CPS) structure (3, 4). Until recently, there were 15 recognized serovars with different geographical distributions (3, 5). Knowing the serovar is important for epidemiological tracking as well as bacterin-based vaccine formulation pertinent to the geographical area (3). The complete CPS biosynthetic loci of these 15 serovars have been determined either specifically (6–8) or as part of whole-genome sequence analysis (9–11). In all 15 serovars except serovar 15, the genes of the CPS biosynthetic locus are divergently transcribed from those of the export locus, *cpxDCBA* (6, 10); in serovar 15, the CPS biosynthetic genes are in the same orientation as, and immediately precede, those of the export locus (7).

**FIG 1:**
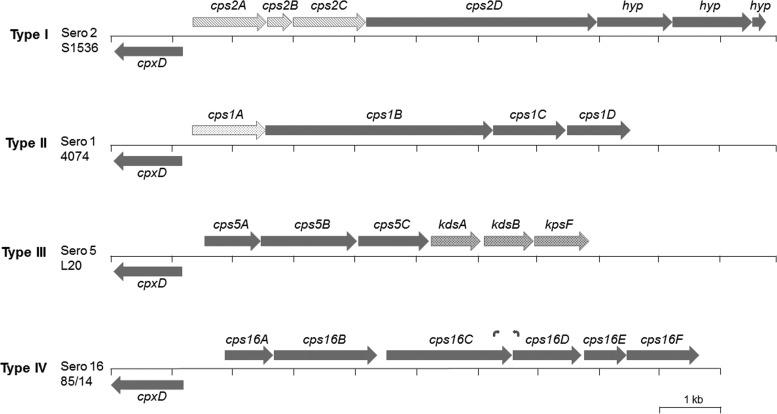
Schematic representation of the A. pleuropneumoniae serovar 16 capsule locus (proposed type IV CPS), and comparison to representative loci for CPS types I to III. The CPS biosynthesis genes for serovar 16 (Sero 16) (*cps16ABCDEF*) are located downstream of and on the opposite strand to the capsule export genes (*cpxDCBA*; only *cpxD* is shown) as in the other loci (note that although not shown, the serovar 15 CPS biosynthetic genes are contiguous with and in the same orientation as the export genes). The relative size and location of each gene are indicated by the sizes and positions of arrows (solid gray or patterned). For type I CPS (serovars 2, 3, 6, 7, 8, 9, 11, and 13), the locus of the serovar 2 reference strain S1536 is shown. All type I CPS loci share three common genes as indicated by the arrows with hatching. For type II CPS (serovars 1, 4, 12, and 15), the locus for the serovar 1 reference strain 4074^T^ is shown. All type II loci share the first gene as indicated by the arrow with the dotted pattern. For type III CPS (serovars 5 and 10), the locus for the serovar 5b reference strain L20 is shown. Type III CPS loci share the three *kds* genes indicated by the arrows with the crosshatched pattern. The newly proposed type IV CPS locus is currently found only in serovar 16, as illustrated by the locus of the reference strain A-85/14. The locations of serovar 16-specific primers are indicated as small curved arrows above *cps16C* (forward primer AP16F) and *cps16D* (reverse primer AP16R).

Xu et al. (10) compared the CPS loci for serovars 1 to 7 and 9 to 13 and showed that they could be grouped into three types (see Fig. 1 for representative loci for each CPS type), in agreement with previous structural studies (4). The loci for serovars 2, 3, 6, 7, 8, 9, 11, and 13 (type I) have a common core, with the first three genes (*cps2ABC*) sharing a high degree of identity and encoding teichoic acid transferase, glycerol transferase, and glycerol-3-phosphate cytidylyltransferase, respectively (8, 10). The type II CPS loci, initially identified in serovars 1, 4, and 12, share the first gene (*cps1A*) encoding a CPS phosphotransferase (10), which is also seen in the subsequently sequenced serovar 14 CPS locus (6). Although not highly conserved at the nucleotide level, *cps15A* (accession no. BAR72992), the first gene in the serovar 15 CPS locus, also encodes a CPS phosphotransferase that shares 62% identity with *cps1A*, suggesting that serovar 15 also produces a type II CPS (7). In the type III capsule loci, seen in serovars 5 and 10, it is the final three genes that are common, encoding 3-deoxy-8-phosphooctulonate synthase, 3-deoxy-manno-octulosonate cytidylyltransferase, and arabinose-5-phosphate isomerase, respectively.

It was recently reported that five Hungarian isolates of A. pleuropneumoniae could not be assigned to any of the known 15 serovars but rather formed a distinct serological group, and it was proposed that they constituted a new serovar of A. pleuropneumoniae—serovar 16 (12). Here, we used whole-genome sequencing to determine the genetic organization of the CPS biosynthesis locus for strain A-85/14, the proposed reference strain for serovar 16. The sequencing data show that the serovar 16 CPS locus is distinct from that of the other known 15 serovars. Furthermore, on the basis of this unique sequence, we have designed primers for specific detection of A. pleuropneumoniae serovar 16.

## RESULTS AND DISCUSSION

The CPS biosynthetic locus for strain A-85/14 was identified as six genes, designated *cps16A* to *cps16F*, transcribed divergently from *cpxD* (Fig. 1). BLASTx revealed that, although similar sequences could be found in other bacterial species (see Table 1 and below), including other members of the Pasteurellaceae family, these six genes are not present in the CPS biosynthetic loci of any other serovar of A. pleuropneumoniae. Furthermore, the arrangement of genes is not similar to that seen in any other bacterial species sequenced thus far.

**TABLE 1:** Homologs of proteins encoded by the predicted CPS biosynthesis locus of A. pleuropneumoniae serovar 16

*Ap*S16 protein^*a*^	Length (no. of aa)	Homologous protein					
		Protein^*b*^	Length (no. of aa)	Bacterium	% ID^*c*^	Accession no.	Known or putative function
Cps16A	261	DcbB	465	A. ureae	82 (91)	WP_005621190	Predicted glycosyltransferase
		DcbB	477	P. multocida	58 (76)	WP_059246061	Predicted glycosyltransferase
		CcbB	309	A. paragallinarum	58 (73)	ACY25522	Predicted glycosyltransferase
			479	G. anatis	57 (73)	WP_021461679	Predicted glycosyltransferase
Cps16B	565	HyaE	662	P. multocida	34 (51)	WP_010906846	Synthesis of hyaluronic acid capsule
			542	G. anatis	34 (52)	WP_065232000	Hypothetical protein
			553	A. paragallinarum	33 (51)	WP_046097317	Hypothetical protein
			415	A. ureae	53 (71)	EFX92827	Hypothetical protein
			379	A. ureae	50 (69)	WP_044024188	Hypothetical protein
Cps16C	678		677	A. seohaensis^*d*^	51 (69)	SDC36622	Predicted glycosyltransferase
		RfbG	664	E. coli	49 (68)	WP_061091032	dTDP-rhamnosyl transferase
Cps16D	367		382	Haemophilus^*e*^	71 (81)	WP_049357969	UDP-galactopyranose mutase
			383	A. pleuropneumoniae	70 (81)	WP_005613015	UDP-galactopyranose mutase
Cps16E	226		217	E. coli	57 (73)	WP_024190280	Hypothetical protein
			206	H. sputorum	31 (47)	WP_007526031	Hypothetical protein
Cps16F	389		390	A. ureae	88 (94)	WP_005621201	UDP-glucose 6-dehydrogenase
			390	G. anatis	67 (14)	WP_065232026	UDP-glucose 6-dehydrogenase
			390	P. multocida	65 (14)	AAK02860	UDP-glucose 6-dehydrogenase

a *Ap*S16 protein, protein encoded by the predicted CPS biosynthesis locus of A. pleuropneumoniae serovar 16.

b Protein names given where available. The matches with highest percent identity are listed first for each protein.

c % ID, percent identity over the matching span. Numbers in parentheses indicate the percentage of amino acids which are similar according to their physiochemical properties over the matching span.

d A. seohaensis, Acinetobacter seohaensis.

e Multiple species of Haemophilus.

The first gene, *cps16A*, encodes a 261-amino-acid (aa) predicted glycosyltransferase that shares greatest identity with the C-terminal half (82% identity for aa 198 to 458 of 465) of DcbB in Actinobacillus ureae (GenBank accession no. WP_005621190). Similar glycosyltransferase proteins are also found in Pasteurella multocida (accession no. WP_059246061), Avibacterium paragallinarum (GenBank accession no. ACY25522), and Gallibacterium anatis (accession no. WP_021461679), though their exact function is not known.

The next gene, *cps16B*, encodes a 565-aa protein with similarity to HyaE (34% identity and 51% positive substitutions over the entire length; GenBank accession no. WP_010906846) involved in synthesis of the hyaluronic acid capsule of P. multocida (13). Again, similar proteins are found in A. paragallinarum (accession no. WP_046097317), and G. anatis (accession no. WP_065232000); with smaller proteins (379 and 415 aa) found in strains of A. ureae (GenBank accession no. WP_044024188 and EFX92827).

The product of *cps16C* is a 678-aa glycosyltransferase that shows extensive similarity to a predicted glycosyltransferase in Acinetobacter seohaensis (GenBank accession no. SDC36622) and RfbG (dTDP-rhamnosyl transferase) from Escherichia coli (GenBank accession no. WP_061091032). There are two predicted rhamnosyl transferase genes (*rfbF* and *rfbN*) present in lipopolysaccharide (LPS) loci of A. pleuropneumoniae serovars 1, 9, and 11 (10), and l-rhamnose was shown to be a component of the LPS O chain in these serovars (4). Structural studies also showed the presence of l-rhamnose in the O chains of serovars 2, 4, 7, and 13 (4, 14), and genes encoding dTDP-4-dehydrorhamnose reductase and dTDP-4-dehydrorhamnose 3,5-epimerase have been identified in these serovars (10). However, this is the first report of l-rhamnose as a predicted component of capsule in A. pleuropneumoniae.

Cps16D is a predicted UDP-galactopyranose mutase that is common to many members of the Pasteurellaceae, including A. pleuropneumoniae serovars 5, 10, and 12 (GenBank accession no. WP_005613015), where it is encoded by the *glf1* gene found in the respective LPS loci (10). A different galactopyranose mutase gene, *glf*, is found in the LPS loci of A. pleuropneumoniae serovars 3, 6, and 8 (10, 15). Although similar in size, the proteins encoded by *glf* and *glf1* share only 43% identity. The enzyme catalyzes the conversion of UDP-galactopyranose to UDP-galactofuranose, which in turn is the precursor of galactofuranose (Gal*f*), a five-membered ring form of galactose found in cell surface glycans in a variety of organisms except mammals (16). Structural studies confirmed the presence of Gal*f* in the LPS O chains of A. pleuropneumoniae serovars 3, 6, 8, 10, 12, 14, and 15 (4, 17, 18), but not in any of the reported CPS structures.

The *cps16E* gene encodes a 226-aa hypothetical protein containing a domain indicative of coenzyme A (CoA)-dependent O-acetylation of a sugar substrate. The protein sequence is most similar (57% identity) to a hypothetical E. coli protein (GenBank accession no. WP_024190280), with the only detected match in the Pasteurellaceae being a protein in Haemophilus sputorum (accession no. WP_007526031) with 31% identity. Structural studies identified O-acetylated sugars in the CPS of serovars 1, 8, 9, 10, and 11 (4); however, the genes predicted to encode acetyltransferases in the CPS loci of serovars 1 (*cps1D*) and 10 (*cps10B*) (10) are distinct from *cps16E*, and no acetyltransferase genes have been identified in the other CPS loci.

The final gene in the locus, *cps16F*, encodes a UDP-glucose 6-dehydrogenase which is most similar (88% identity) to that found in A. ureae (GenBank accession no. WP_005621201), with proteins in P. multocida and G. anatis (accession no. AAK02860 and WP_065232026, respectively) sharing less than 70% identity. This enzyme catalyzes the conversion of UDP-glucose to UDP-glucuronic acid, the substrate for generation of various surface structures, including CPS, LPS, and exopolysaccharides in a variety of bacterial species (19).

**FIG 2:**
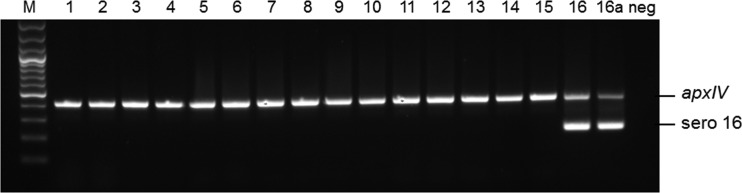
Verification of specificity of primers for molecular identification of A. pleuropneumoniae serovar 16. An *apxIV* (418-bp) amplicon is detected in all 16 serovar reference strains; the serovar 16-specific amplicon (212 bp) is detected only in the serovar 16 reference strain A-85/14 and the representative clinical isolate, 151/16. Lane M contains molecular size markers (100-bp ladder). Lanes 1 to 16 contain the following strains: 1, 4074^T^; 2, S1536; 3, S1421; 4, M62; 5, L20; 6, Femo; 7, WF83; 8, 405; 9, CVJ13261; 10, D13039; 11, 56153; 12, 8329; 13, N-273; 14, 3906; 15, HS143; 16, A-85/14. Lane 16a contains serovar 16 strain 151/16, as representative of all serovar 16 clinical isolates tested, and lane neg contains no DNA and is a negative control.

For diagnostic purposes, we designed primers to amplify a unique 212-bp fragment spanning the 3′ end of *cps16C* and the 5′ end of *cps16D* (Fig. 1). When used in conjunction with previously designed *apxIV* primers for detection of all serovars of A. pleuropneumoniae (8), the serovar 16 band was successfully amplified (along with *apxIV*) from the original five isolates (12) as well as two new Hungarian isolates, but the reference strains of A. pleuropneumoniae serovars 1 to 15 produced only the *apxIV* amplicon (Fig. 2). Further testing of clinical isolates of A. pleuropneumoniae and other bacteria associated with pigs, including publicly available genomes, confirmed the specificity of the primers for serovar 16. Although currently identified only in Hungary, the serovar 16-specific primers designed here will be important for wider epidemiological monitoring of the distribution of this newly identified serovar. The size of the amplicon generated should allow these primers to be incorporated into existing multiplex PCRs (8, 20). We did not evaluate the use of the serovar 16 primers in quantitative PCR (qPCR).

In conclusion, we have identified the predicted capsule locus of A. pleuropneumoniae serovar 16 and confirmed that it is unlike that in any other serovar. Sharing no common genes with any of the type I to III CPS loci previously identified in A. pleuropneumoniae, it represents a new type IV locus. The combination of DNA sequence data (this study) and serology (12) show the existence of a sixteenth serovar—designated serovar 16—of A. pleuropneumoniae. Structural analysis of the serovar 16 capsule is under way.

## MATERIALS AND METHODS

Actinobacillus pleuropneumoniae strains used in this study were as follows: the recently proposed serovar 16 reference strain A-85/14 plus the four other strains described by Sárközi et al. (12) and two further 2016 Hungarian isolates (151/16 and 228/16) identified as serovar 16 by serology; serovar 1 to 15 reference strains, i.e., 4074^T^, 1536, S1421, M62, L20, Femø, WF83, 405, CVJ13261, D13039, 56153, 8328, N-273, 3906, and HS143, respectively (see also reference 21 for further details); and a collection of 68 clinical isolates (5 isolates each of serovars 1 to 8, 9/11, 10, 12, 14, and 15 and 3 isolates of serovar 13). In addition, we tested 31 strains of other bacterial species associated with pigs that have been described previously (8, 21). These strains included the type strains for Actinobacillus suis (CCM 5586^T^), Actinobacillus minor (NM305^T^ = CCUG 38923), Actinobacillus porcinus (NM319^T^ = CCUG 38924), Actinobacillus indolicus (46KC2^T^ = CCUG 39029), Haemophilus parasuis (1374^T^ = NCTC 4557), and strain CCUG 46996 for “Actinobacillus porcitonsillarum.” Clinical isolates of Bordetella bronchiseptica and Mycoplasma hyopneumoniae were obtained from the Royal Veterinary College Diagnostic Laboratory and identified to the species level by standard methods (22, 23). Pasteurella multocida porcine clinical isolates were from clonal complex 13 as determined by multilocus sequence typing (24). Furthermore, we performed virtual PCRs with publically available genomes for Actinobacillus ureae (3520/59^T^ = NCTC 10219), M. hyopneumoniae (J^T^ = NCTC 10110), Streptococcus suis (S735^T^ = NCTC 10234), P. multocida (*n* = 61 including W-9217^T^ = NCTC 10322), B. bronchiseptica (*n* = 68), and H. parasuis (*n* = 212 including 1374^T^ = NCTC 4557 [25]).

Shotgun whole-genome sequence data for A. pleuropneumoniae strain A-85/14 was generated and assembled by the Microbes NG Sequencing Facility (www.microbesng.uk). The contig containing the capsule locus was identified by BLASTn, using the sequence of the *cpxD* gene (GenBank accession no. AIA09380) from the capsule export locus, which is common to all serovars.

Serovar 16-specific primers, AP16F (F stands for forward) (TTACTCACTTGGGCTAGGGATAG) and AP16R (R stands for reverse) (ACCAGCAATATGATTACGCCC) were designed to amplify a 212-bp fragment as shown in Fig. 1. These primers were initially tested for specificity using genomic DNA from the 16 reference strains (including strain A-85/14). Subsequently, the primers were combined with apxIVA1 (TTATCCGAACTTTGGTTTAGCC) and apxIVA3 (CATATTTGATAAAACCATCCGTC) that amplify a 418-bp fragment of the species-specific *apxIV* gene of A. pleuropneumoniae (8) and further evaluated for specificity using the other 101 isolates, including the negative controls. Amplification was performed using the Qiagen Fast Cycling PCR kit, according to the manufacturer's instructions (Qiagen). Each reaction mixture contained 10 μl Qiagen Fast Cycling PCR master mix, 2 μl CoralLoad dye, 1 μl genomic DNA, each primer at a final concentration of 0.5 μM, and water to a final volume of 20 μl. Cycling conditions included an initial 5-min activation at 95°C, followed by 30 cycles of PCR, with 1 cycle consisting of denaturation at 96°C for 5 s, annealing at 60°C for 5 s, and extension at 68°C for 10 s, followed by a final extension at 72°C for 1 min.

### Accession number(s).

The sequence of the complete capsule locus for serovar 16 (strain A-85/14) has been deposited in GenBank under accession number KX907602.
